# Translational Control in *p53* Expression: The Role of 5′-Terminal Region of p53 mRNA

**DOI:** 10.3390/ijms20215382

**Published:** 2019-10-29

**Authors:** Agata Swiatkowska, Mariola Dutkiewicz, Paulina Zydowicz-Machtel, Joanna Szpotkowska, Damian M. Janecki, Jerzy Ciesiołka

**Affiliations:** Institute of Bioorganic Chemistry, Polish Academy of Sciences, Noskowskiego 12/14, 61-704 Poznan, Polandjsroka@ibch.poznan.pl (J.S.); djanecki@ibch.poznan.pl (D.M.J.)

**Keywords:** p53 expression, p53 mRNA, 5′ untranslated region, 5′ non-coding region, 5′UTR, transcription, translation initiation, translation regulation

## Abstract

In this review, the latest research concerning the structure and function of the 5′-terminal region of p53 mRNA was discussed. Special attention was focused on defined structural motifs which are present in this region, as well as their conservation and plausible functional role in translation. It is known that the length of the 5′-terminal region and the structural environment of initiation codons can strongly modulate translation initiation. The ability of this region of p53 mRNA to bind protein factors was also described with special emphasis on general principles that govern, such RNA-protein interactions. The structural alterations within the 5′-terminal region of p53 mRNA and proteins that bind to this region have a strong impact on the rate of mRNA scanning and on translation efficiency in in vitro assays, in selected cell lines, and under stress conditions. Thus, the structural features of the 5′-terminal region of p53 mRNA seem to be very important for translation and for translation regulation mechanisms. Finally, we suggested topics that, in our opinion, should be further explored for better understanding of the mechanisms of the *p53* gene expression regulation at the translational level.

## 1. Introduction

Recent decades of intense research have clearly shown that the multi-level regulation of gene expression has a major impact on the eventual level of protein synthesis. Since the p53 protein is one of the key players in regulating a plethora of processes in the cell by activating genes that often have opposite functions, it is considered to be the cell homeostasis guardian [1]. The level, as well as the activity of the p53 protein, is tightly regulated at many layers [2,3,4,5]. It has emerged that the first level of such regulation concerns the transcription process. It has been demonstrated that several factors are able to specifically bind the p53 DNA promoter and regulate p53 transcription [6]. However, other data have revealed that regulation at the post-transcriptional and translational levels also plays a pivotal role in the *p53* gene expression modulation [7,8,9,10]. Very recently, the role of the p53 mRNA as an integral part of the cellular stress response was reviewed [11]. 

Importantly, the ability of RNAs to form intramolecular interactions gives these molecules a higher structural flexibility and plasticity compared to DNA, which adopts a double-helix conformation. This results in more possibilities of structural rearrangements and various interactions with proteins and other RNA molecules [12,13]. Thus, the function of RNA often depends on its 3D structure and ability to acquire distinct conformations [14]. These features of RNA have been nicely illustrated during the assembly of spliceosomes and pre-mRNA splicing [15]. Also, the translation of mRNAs depends on higher-order structures of these molecules, and in particular, their 5′UTRs have been shown to participate in such regulation [16,17,18]. These regions play a crucial role in the translation initiation, and most mechanisms of the translational control affect the rate of initiation [18,19,20]. 

It has been shown that the 5′-terminal region of p53 mRNA is important for translation, particularly when the p53 level must be quickly elevated in response to stress conditions [21]. An increase in translation efficiency results from the activity of two internal ribosomal entry sites (IRESes) [22,23]. Although these IRESes have not been thoroughly characterized, their presence in the p53 mRNA is widely accepted (reviewed in [7,11,17,24]). Moreover, several protein factors influence the *p53* gene expression via binding to the 5′ terminus of p53 mRNA [11,24]. We recently showed that the 5′ terminus of p53 mRNA folds into several characteristic structural motifs [21,25,26]. These motifs presumably play a role as docking platforms for proteins to regulate the *p53* gene expression at the translational level. Data from our recent work also confirmed a strong correlation between the structural folding of the 5′-terminal region of p53 mRNA and the kinetics of the translation initiation process [27]. 

In this review, the latest research concerning the structure and function of the 5′-terminal region of p53 mRNA was discussed. Special attention was focused on defined structural motifs which are present in this region, as well as their conservation and plausible functional role in translation. We reviewed the available information on interactions of this mRNA region with *trans*-acting proteins which are able to impact the p53 translation process, particularly under stress conditions. We also shed more light on how structural changes within the 5′-terminus of p53 mRNA influence translation efficiency. Finally, we proposed topics that, in our opinion, should be further explored for better understanding of the mechanisms of the *p53* gene expression regulation at the translational level.

## 2. Transcription Initiation Sites and Secondary Structure of the 5′-Terminal Region of p53 mRNA

In an earlier study, complex secondary structures in the 5′UTRs were mostly correlated with inhibition of translation [28]. Currently, computer modelling of RNA structures, combined with recent RNA probing methodologies, allow for better characterization of RNA secondary structure in vitro [29,30,31] and in vivo [32]. Thereby, a deeper understanding of the multi-functions of these regions can be obtained. The determination of transcription initiation sites of the *p53* gene and elucidation of the secondary structure of the 5′-terminal region of p53 mRNA have allowed for studies aimed at characterizing RNA structural motifs, which are important for the functioning of this mRNA region. 

The promoter regions of the human *TP53* gene have been determined by primer extension assay combined with S1 nuclease treatment and northern blot analysis. At least three transcription initiation sites for mRNA templates encoding full-length p53 protein and its major isoform, Δ40p53, have been found: P0, P1, and P1′ [33]. All of these sites are located in the first exon. Near the 5′ end of the *TP53* gene promoter, P0 is located, which corresponds to the 5′UTR of approximately 250 nucleotides in length [33,34]. The region termed as a P1 promoter site is located around the 114 [35] or 117 [33] position, upstream of the 3′ end of the exon 1. It has been observed that transcripts beginning at the P1 site are heterogeneous in length. However, a majority of transcripts comprise the 5′UTR consisting of approximately 140 nucleotides [33]. Additionally, transcripts with the 5′UTR of 190 nucleotides in length have also been detected, which reflect the promoter activity located between P0 and P1 initiation sites. This region has been termed as the P1′ promoter site [33,35]. 

Interestingly, tissue-specific synthesis of different p53 transcripts have been observed [33]. The P0-initated transcripts account for approximately 50% of the total p53 mRNA in normal human tissues. Transcripts beginning at the P1 site have been detected in non-tumorigenic, immortalized cell lines, such as BEAS-2B and MSK-Leuk1, and in several tumorigenic cell lines. These cells are derived from various organs: The larynx, oral cavity, breast, liver, and lungs. Additionally, in some tissues, such as the spleen and thymus, all types of the p53 transcripts have been detected, which reflects the heterogeneous nature of the *TP53* gene transcription [33]. 

Recently, we determined transcription initiation sites for the mouse *Trp53* gene by the 5′RACE method [36]. The most frequent transcripts isolated from the embryos, liver and thymus, termed as mRNA-122, contain the 5′UTR comprising approximately 122 nucleotides. This is in line with earlier observations concerning the identification of mouse transcription promoter regions [37]. Interestingly, the comparison of the 5′-terminal sequences of mouse mRNA-122 and of human P1-initiated p53 mRNA revealed that the transcripts differ only by a single nucleotide in spite of a substantially different length of their 5′UTRs [36]. We also detected longer transcripts, termed as mRNA-247, which comprise the 5′UTR region of approximately 247 nucleotides in length. These transcripts have been observed in mouse embryos, and additionally in the brain, heart and spinal cord tissues [36]. 

The 5′-terminal region of the guinea pig p53 transcript has been analyzed by sequencing RNA isolated from spleen tissue [38]. The comparison of the guinea pig 5′UTR of 233 nucleotides in length, while the corresponding regions in mammalians revealed about 60–70% similarity in sequence composition despite differences in the length of these regions [38]. However, no exact transcription initiation site(s) of the guinea pig *p53* gene has/have been determined so far. 

**Figure 1:**
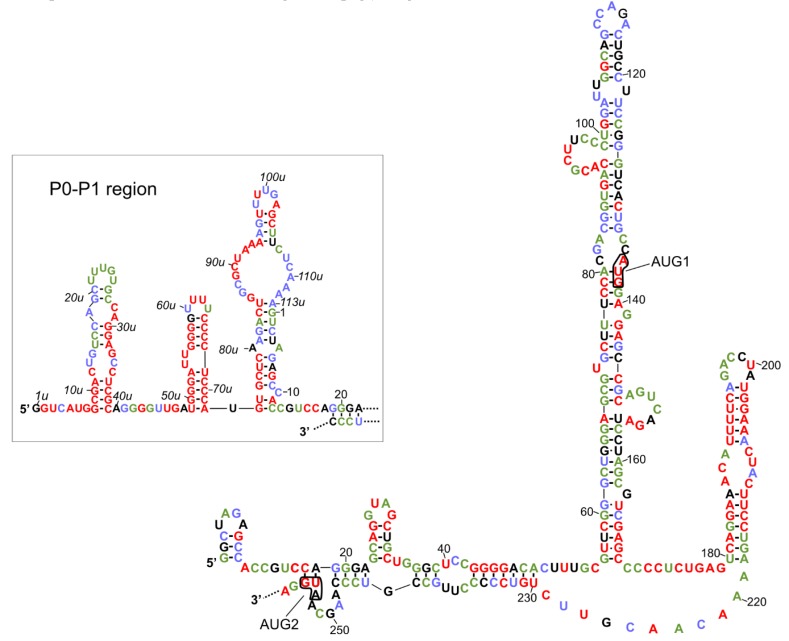
Secondary structure models of the 5′ terminal regions of p53 mRNA that begin at P1 and P0 (in the box) transcription initiation sites. The level of each nucleotide conservation is marked based on the alignment of p53 mRNA sequences derived from eleven different species (Figure 2 in the citation [36]). The nucleotides are colored according to the percentage of their conservation (red, 100%; green, 80–99%; blue, 60–79%).

The secondary structure models of variants of the 5′-terminal regions of human p53 mRNA have been proposed [25,26]. Two identical hairpin motifs were present in the 5′-terminal regions of both P1- and P0-initiated transcripts, P1-Δ40p53 and P0-Δ40p53 mRNAs (Figure 1). The first motif, which consists of nucleotides spanning G56 and C169, folded into a large thermodynamically stable hairpin domain in which AUG1 for full-length p53 was embedded. The second motif, involving the U180-A218 region, folded into a smaller hairpin, which has been shown to be recognized by several proteins, for example, HDM2, PTB and hnRNP C1/C2 ([25,39,40], see also the next chapter). Additionally, the 5′-proximal region of P0-initiated transcript was folded into three hairpin structures (Figure 1, the inset; based on the data from [26]). Importantly, both translation initiation codons, AUG1 for full-length p53 and AUG2 for Δ40p53 isoform, were located in the helix-bulge junctions, and the A residues were unpaired. It has been shown that the approximately five-nucleotide-long region surrounding the start codon displays a significant lack of structure for genes with high translation efficiencies and is structured in those with low efficiencies [13,41,42]. The specific structural location of the initiation codons in p53 mRNA seems to be very important, since changes in the structural environment of AUG1 in the model p53 mRNA constructs have a large influence on ribosomal scanning and translation efficacy [27]. 

The secondary structure model of the 5′-terminus of human p53 mRNA was compared with the nucleotide conservation of this region in various organisms (Figure 1). This comparison was based on the earlier presented alignment of the 5′-terminal regions of p53 mRNAs derived from 11 different species (Figure 2 in [36]). This data showed which structural elements are mostly conserved due to the important role in the folding of this RNA region or due to functional interactions with other factors, likely proteins. It was found that the U180-A218 region, which is folded into a hairpin, is highly conserved. In particular, the upper six base pairs of hairpin’s stem are characterized by almost 100% conservation. Since it has been observed that the U180-A218 hairpin is a binding platform for some proteins, we envisaged that this structural feature of the 5′-terminal regions of p53 mRNAs might be involved in the regulatory process of p53 expression at the translational level. 

The second conserved region comprises four guanosine residues in positions from 43 to 46 which are engaged in the formation of a double-stranded stem structure. Interestingly, another poly(G) tract involved in base paired stem, located in positions 56u to 58u in the P0-P1 region, is also characterized by 100% conservation (Figure 1, the inset). It is likely that poly(G) tracts are necessary to maintain the proper folding of global RNA structure, since they are mostly engaged in the formation of double-stranded stems. 

The hairpin composed of nucleotides in positions 49u to 72u, which is present in the 5′-proximal region of P0-initiated transcript, has emerged as a highly conserved structural feature. Only two nucleotides in its apical loop, and one nucleotide close to this loop, seem to be dispensable. Possibly, this hairpin is recognized by *trans*-acting protein factors, which might influence p53 translation. The 8u-39u stem-loop structure, located in close proximity to the 5′ end of P0-initiated transcript, was also partly conserved. Particularly, the bottom part of a double-stranded stem and the right side of this hairpin are characterized by a high level of sequence preservation (Figure 1, the inset). Interestingly, analysis of nucleotide conservation among different species revealed that the P0–P1 region was mostly highly preserved. It has been observed that the P0–P1 region has a strong inhibitory effect on the p53 translation efficiency [26,27,33]. Although the biological role of this region has not yet been fully understood, it can be anticipated that the maintenance of the structural features of P0–P1 region of mRNA is crucial for its function.

**Figure 2:**
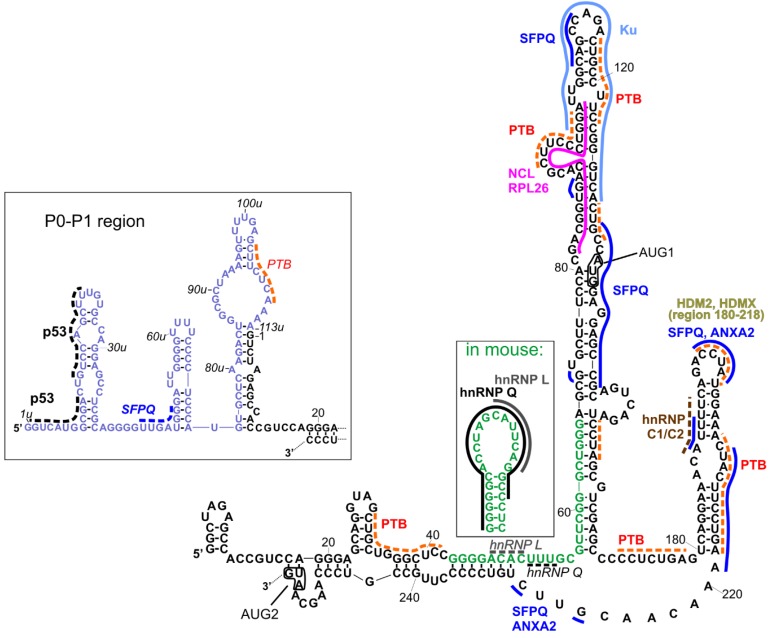
Interactions with proteins that were indicated for variants of 5′UTR of p53 mRNA. Black fonts represent the RNA sequence of human 5′UTR of P1-Δ40p53 mRNA. Blue fonts indicate the region between P0 and P1 transcription start sites present at the 5′ end of P0-p53 mRNA. Green fonts show a differently folded fragment of analogous sequence present in mouse p53-mRNA. Names of proteins, written in bold, pertain to the established interactions with one of 5′UTR-p53 variants. The particular site of such interactions is indicated by solid line in the same color as the name of the protein. In the event that the particular site of the interaction is not confirmed but only predicted, the line is dotted. Italics depict interactions which were not indicated for human mRNA variant and are proposed in analogy to mouse. For some proteins, only wider regions of interactions were investigated, and there are no lines indicating their interaction sites. Translation start sites are depicted in boxes and named AUG1 and AUG2. Detailed description is provided in the text.

Finally, nucleotides of the G56–C169 hairpin domain, which are characteristic of both 5′UTRs of P1-Δ40p53 and P0-Δ40p53 mRNAs, were less conserved. Moreover, in the mouse p53 mRNA, this hairpin was shorter and its bottom segment was unfolded [36]. The 5′-proximal stretch of the unfolded segment formed another hairpin (see the inset in Figure 2), while the 3′-proximal stretch remained single-stranded. Thus, the upper part of the G56-C169 hairpin, found in human p53 mRNA, seems to be functionally more important. 

## 3. Interactions of the 5′-Terminal Region of p53 mRNA with Proteins

For decades, research has focused on linear RNA sequence motifs bound by proteins that control post-transcriptional processes [43,44]. Nowadays, the importance of the structural context of short linear motifs in RNA recognition is emphasized, including spaced motifs, flanking nucleotide stretches, and preferences toward particular RNA structures [45]. Thus, in current studies aimed at accessing the regulatory role of proteins that bind to the 5′-terminal region of p53 mRNA, whenever possible, the structural context of the protein binding site needs to be considered to better understand these interactions. 

Figure 2 represents a compilation of the secondary structure models of the 5′UTRs of two p53 mRNA variants and their established interactions with proteins [11,26]. It has been shown that both regions can be used in an internal ribosome entry site (IRES)-dependent manner, especially under stress conditions [22,23]. The interacting proteins may play a role of IRES *trans*-acting factors (ITAFs), thereby regulating their function [24]. Other proteins can also be involved in the regulation of standard, cap-dependent translation, or they can influence mRNA stability, trafficking, or other processes in which they participate. 

To the best of our knowledge, most of the interactions shown in Figure 2 have been proposed for human p53 mRNA variants, while some interactions have been proposed for human and mouse homologs, and two of interactions were proposed only for the mouse homolog. As far as the structural context is concerned, interactions with heterogenous nuclear ribonucleoproteins (hnRNP), hnRNP Q and hnRNP L, established only for mouse 5′UTR ([46,47], respectively), are particularly interesting. The region of interactions with hnRNP L was placed within an apical loop of a hairpin, and hnRNP Q interacted with almost all nucleotides of this hairpin, whose structure has been proposed only for mouse 5′UTR (Figure 2, the inset). In the human p53 mRNA, the respective sequence (green fonts in Figure 2) was partially engaged in the double-stranded stem, a single-stranded linker, and a basal, double-stranded stem of a long hairpin domain containing the AUG1 initiation codon. Thus, the question of whether the secondary structure of that hairpin plays an important role in these interactions was raised, since they have been found only for mouse and not for human mRNA. However, in Figure 2, we depicted possible sites of the hypothetical interactions of hnRNP L and hnRNP Q with the human mRNA analog (italics and dotted lines), taking into account the sequence preferences of those proteins (CA-rich sequences for hnRNP L; AU-rich/A-rich/U-rich sequences for hnRNP Q). Such preferences have been described in [45] based on comparison of several known interaction sites deposited in databases, such as ATtRACT [48] and RBPmap [49], and the latest results of high-throughput experiments [50]. 

In the P0-P1 region, only one kind of interaction—self-regulatory interactions with p53—has been evidenced so far [51]. The p53 binding sites have been proposed close to the 5′-terminus in a region which corresponds to the recognition sites within the *TP53* gene promoter [52]. Two other possible protein binding sites in the P0-P1 region can be proposed for SFPQ (splicing factor proline and glutamine rich) (dark blue dotted line) and PTB (polypyrimidine track binding protein) (orange dotted line) using the ATTRACT database. The interactions with SFPQ have been mapped in multiple sites within long 5′UTR (Figure 2, dark blue solid line). 

The PTB protein has been mapped to the p53 mRNA upstream and downstream AUG1, though with different binding affinities. Stronger binding occurs in the region downstream AUG1 and it enhances translation of Δ40p53 [53]. More detailed research, with the use of foot-printing and toe-printing methods, has shown that PTB binds to multiple sites along p53 5′UTR [40]. Taking into account these data, as well as PTB binding preferences according to the ATTRACT database, we proposed several PTB binding sites in p53 5′UTR, which are shown in Figure 2 (orange dotted lines). 

The SFPQ protein, also known as PSF, has been shown to be strongly associated with the regulation of translation rate for full-length p53 and Δ40p53 isoform [54]. In the foot-printing experiment, multiple RNA-protein contact sites were found. They comprise the neighborhood of AUG1 codon, nucleotides in positions 108 and 112, the U180-A218 region, and the following linker region (in Figure 2, these sites are indicated with dark blue lines). Some of them are shared with another protein interacting with p53 mRNA, Annexin A2 [54], including the sites in the HDM2 hairpin and in the following linker region, which are annotated as ANXA2 in Figure 2. 

Nucleolin and ribosomal protein RLP26 are other protein partners which regulate the translation of p53 mRNA by competing for common binding site on the mRNA and by interacting with each other [55,56]. Their binding sites were mapped to the proposed region of long-range interactions beween 5′ and 3′ UTRs of p53 mRNA and they are depicted in Figure 2 (pink/fuhsja line). However, the 5′–3′ RNA–RNA interaction itself needs to be confirmed yet. 

The Ku protein binds to the apical loop of a large hairpin domain, in which the AUG1 codon is embedded [57]. The protein represses p53 synthesis and p53-mediated apoptosis, but Ku-mediated translational repression is relieved after genotoxic stress [57]. 

It has been found that hnRNP C1/C2 binds in the double-stranded region of the U180-A218 hairpin [58] with the contact site at U193 [59]. The brown dotted line in Figure 2 indicates the predicted interaction of this protein with p53 5′UTR by comparison to the data presented for hnRNPC in [45] (motif AUUUU), and predicted by the ATTRACT application (motif AUUUUU). 

HDMX (human/murine double minute 4) and its homolog HDM2/MDM2 (human/murine double minute 2) bind the same region within p53 mRNA, but with different specificity and functional consequences [39,60]. These proteins were mapped to the U180-A218 hairpin [61]. HDMX binds the nascent p53 mRNA to promote a conformation that supports the p53 mRNA-HDM2 interaction and the induction of p53 synthesis. 

There are other proteins interacting with the p53 mRNA for which no specific binding sites have been determined or even predicted besides being positioned upstream (group 1) or downstream (group 2) the AUG1 codon. These proteins are engaged in the regulation of translation from one or both p53 IRESes. In group 1, next to the above-mentioned proteins (p53, SFPQ, PTB, hnRNP L, hnRNP Q, NCL, RPL26, and Ku), there are also proteins RNPC1 [62], Pdcd4 [63,64], DAP5 [65], TCP80, and RHA [66,67]. Group 2 consists of the proteins described above (HDMX, HMDM2, PTB, hnRNPC1/C2, SFPQ, and Annexin A2), and additionally, of not localized proteins APP [68] and DAP5 [65]. There is another protein that has been found to interact with the 5′UTR of p53 mRNA, SMAR 1 (Scaffold/matrix-associated region-binding protein 1), but no detailed mapping information has been evidenced so far. This protein is involved in p53 translation upon stress conditions generated by glucose starvation [69]. 

## 4. The Role of the 5′-Terminal Region of p53 mRNA in Translation

### 4.1. Influence of the Secondary Structure of the 5′-Terminal Region of p53 mRNA on Translation 

It has been shown that secondary structure of the 5′-terminal region of mRNA may significantly influence protein translation [70]. In particular, the presence of structural motifs of high thermodynamic stability in this region is undesirable since they must be first unfolded by numerous helicases, which may delay the scanning process [71,72]. Interestingly, increasing the 5′ leader length does not necessarily lead to reduced translational efficiency [73], emphasizing the importance of the structure of this region for translation initiation. 

An excellent example showing that the secondary structure of the 5′-terminal region of mRNA affects the initiation and efficiency of translation is offered by p53 mRNA. Initially, the secondary structure predictions of this region have revealed the presence of a stable, highly ordered, secondary structure that could suppress translation [55]. These predictions were experimentally verified and the secondary structure models of major variants of the 5′-terminal regions of p53 mRNA were proposed ([25,26] and Figure 1). 

**Figure 3:**
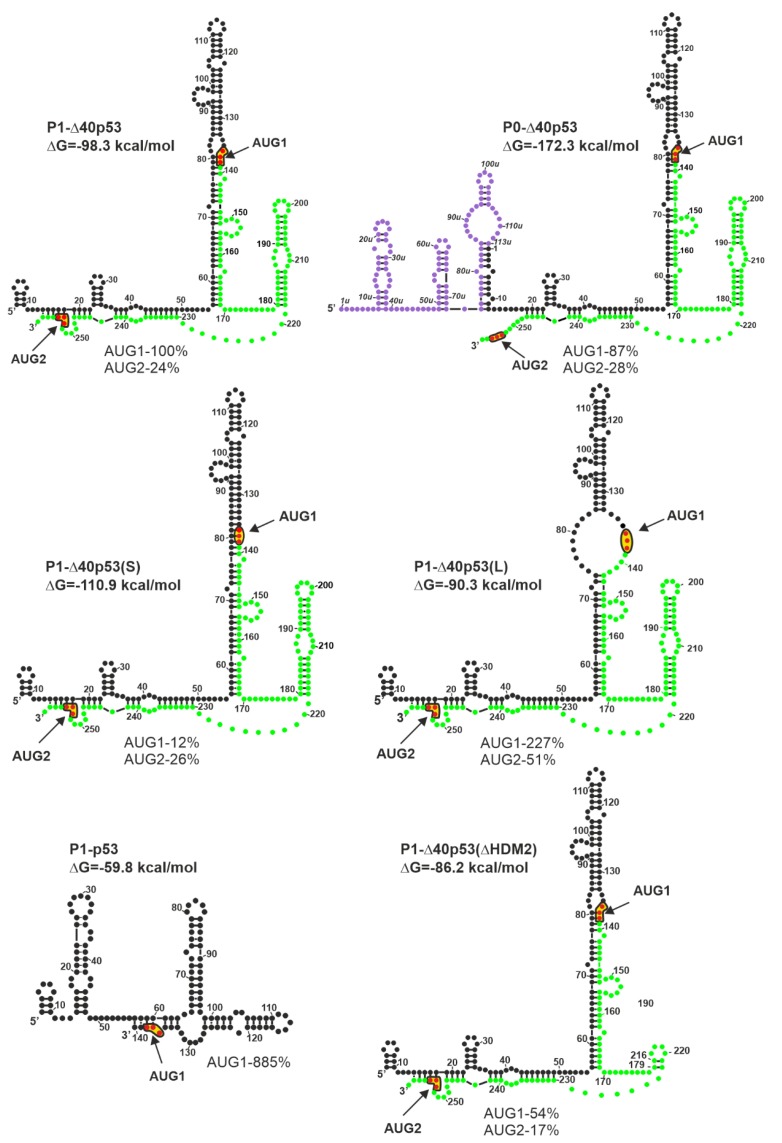
Different length and structure of the 5′-terminal region of p53 mRNA influence the translation efficiency of p53 and Δ40p53 (modified from [27]). Colors denote parts of the 5′-terminal region of model p53 mRNAs: Violet–the region between P0 and P1 transcription promoters; black–the 5′ untranslated region downstream P1 promoter; green–p53 open reading frame (ORF). The predicted ΔG value (kcal/mol) for each 5′UTR is indicated. The data given as percentage illustrate approximate values of maximal translation efficiency for each construct from AUG1 and/or AUG2 normalized to the value obtained for translation of model P1-40p53 mRNA from the AUG1 initiation codon.

It has been found that the structure of the 5′ non-coding region of p53 mRNA has a significant impact on the p53 protein amount that is generated in vitro in rabbit reticulocyte lysate ([27] and Figure 3). Since cell lysates are deprived of most regulatory proteins [74], such conditions are well-suited to evaluate the effect of RNA structure on translation. In fact, the occurrence of the AUG1 initiation codon in a large structural domain of the hairpin-type causes low efficiency of p53 synthesis [27]. The translation efficiency of model P1-p53 mRNA, which lacks the structural element from AUG1, was found to be eight-fold higher than that of P1-Δ40p53 harboring the hairpin, despite the same nucleotide sequence of both 5′UTRs (Figure 3). Moreover, the translation efficiency initiated from the AUG1 codon of P0-Δ40p53 mRNA, which has a longer 5′-terminal region that is folded into three hairpin motifs, was lower by 13% in comparison to that of the P1-Δ40p53 mRNA. 

However, the absence of some secondary structure motifs in 5′ non-coding regions of mRNAs is not always beneficial for protein synthesis. Some structural elements play a crucial role in the initiation process and their interactions with protein factors enhance translation efficiency. In the case of P1-Δ40p53(ΔHDM2) mRNA, the removal of the HDM2 hairpin resulted in the reduction of translation efficiency initiated from the AUG2 codon by about 7% (Figure 3). Surprisingly, the translation initiated from AUG1 codon was also decreased, by almost 50%, which further indicates the important regulatory role of the hairpin with AUG1 codon. 

The structural environment of AUG initiation codons has a significant impact on translation efficiency. The translation efficiency of the P1-Δ40p53(S) mRNA initiated from AUG1 was much lower than that of the P1-Δ40p53 variant. This was the result of the start codon localization in a double-stranded secondary structure, which limited its availability for the translation initiation machinery. As expected, the translation efficiency initiated from AUG1 located in the single-stranded RNA region in variant P1-Δ40p53(L) was higher than that of wild-type P1-Δ40p53 mRNA (Figure 3). An extremely interesting example was the increase of translation efficiency occurring from the AUG2 codon, by about 30%, in the case of variant P1-Δ40p53(L) compared to the translation of P1-Δ40p53 mRNA. It confirmed that the stronger attachment of the ribosome due to the single-stranded region not only facilitates protein synthesis from AUG1 for p53 protein, but also from AUG2 for Δ40p53 isoform (Figure 3 and [27]). 

### 4.2. Stress-Dependent Translational Control of p53 and Its Isoforms Through the 5′-terminal Region of p53 mRNA

It is well-established that the p53 protein becomes activated in response to various stress factors in the cell (Table 1). However, cellular stress may not only affect the phosphorylation state of p53 protein, but also the translation efficiency of the p53 mRNA. An example of stress-response translational activation of p53 was reported in 2005 by Takagi et al. [55]. RNA-pull down assays performed in cell lines have shown that RPL26 is preferentially precipitated by the 5′UTR of p53 mRNA after ionizing irradiation (IR) that leads to DNA damage. The translation of p53 mRNA is enhanced as a consequence of the RPL26 binding and IR stress. The underlying mechanism assumes that stress activates RPL26 and elevates its affinity to the 5′UTR of p53 mRNA. In turn, the interaction alters the distribution of the mRNA on polysomes and can enhance p53 translation [55]. On the other hand, nucleolin, which also binds to the 5′UTR of p53 mRNA, negatively regulates the p53 translation under DNA damage conditions [55]. 

Another example of the p53 translational induction after DNA damage concerns subjection of the cells to etoposide. It was found that the p53 level was increased after etoposide treatment. Presumably, DNA damage turned on the cap-independent translation due to the presence of an IRES element in the 5′-terminal region of p53 mRNA, enabling the IRES-mediated translation [22,23]. An exceptional observation has been made after etoposide treatment of the cells harboring the mutation in *DKC1* (Dyskerin Pseudouridine Synthase 1) gene, a mutation that occurs in X-linked dyskeratosis congenita syndrome [75]. DNA damage triggered by etoposide caused a decrease of IRES-dependent translation of p53 mRNA. On the contrary, the IRES-dependent translation of p53 mRNA was increased in cells without mutation. IRES-dependent translation was also suppressed in mutated cells during oncogenic-induced senescence (OIS), an anti-cancer pathway, resulting in a decreased overall level of p53. This suggested that deregulation of p53 translational control may contribute to increased cancer susceptibility [75].

Etoposide has also been shown to influence the synthesis of mouse p53 protein. It has been found that under this stress agent, the hnRNP Q protein becomes accumulated and binds to the murine 5′UTR of p53 mRNA causing its translational activation [46]. 

The observations of Christian et al. have also supported the suggestion that translation of p53 mRNA might be regulated by stress conditions in the cell [58]. DNA damaging drugs, cis-platin and actinomycin D, elevate the synthesis of p53 by influencing its mRNA translation. The drugs likely change the phosphorylation state of hnRNPC1/C2 in the cell, thus enhancing its affinity to the 5′UTR of p53 mRNA. In fact, this protein binds to the hairpin-type structure present in this region of mRNA. This reveals that this structure may be a part of the machinery that controls p53 expression when transcription is disturbed due to higher levels of p53 after the transcriptional inhibition induced by actinomycin D [58].

Another genotoxic reagent causing DNA damage is doxorubicin. The subjection of cells to this drug enhances the synthesis of p53 in a HDM2-dependent manner [61]. HDM2 is a well-known p53 antagonist that ubiquitinates p53 and directs the protein to degradation [78]. However, it also positively regulates the translation of p53 mRNA by binding to its secondary structure element after phosphorylation performed by ATM-kinase due to stress. HDM2 phosphorylation changes its conformation, which elevates its affinity to p53 mRNA and, at the same time, impairs HDM2-dependent degradation of p53 [61].

It has been shown that another kind of cellular stress, endoplasmic reticulum (ER) stress, also influences the translation of p53 mRNA [76]. Exposure of the cells to thapsigargin, causing ER stress, promotes PERK-dependent induction of Δ40p53 synthesis. In this process, the first 120 nucleotides downstream the AUG1 codon are involved. In turn, an increased level of Δ40p53 induces G2 arrest indicating the extremely important role of the mRNA secondary structure for ER stress-induced G2 arrest [76].

The translation of the p53 mRNA may also be controlled by the starvation stress, which is induced by the presence or absence of glucose in the cell [77]. It has been established that the SMAR1 protein is important for IRES-dependent Δ40p53 and p53 synthesis. It has been shown that in mammalian cell lines, the SMAR1 protein becomes accumulated in cytoplasm under glucose deprivation conditions. In turn, the SMAR1 protein binds to the IRES structure located in the 5′UTR of p53 mRNA and activates the translation of Δ40p53 and p53 [77]. During starvation, the levels of both proteins increase. As a consequence, the 14-3-3σ mRNA encoding tumor suppressor protein becomes elevated, suggesting the critical role of p53 translational control in cancer prevention. Thus, it is clear that mRNA-binding proteins play an important role in p53 expression during cellular stress by modifying both the translation and mRNA stability. Moreover, the secondary structure elements of the 5′-terminal region of p53 mRNA play a pivotal role in the translational control. This implies that the translational control of p53 levels in cells is of great importance, especially in the terms of tumor suppression.

### 4.3. Functional Importance of the Sequence and the Structure of the 5′-Terminal Region of p53 mRNA

The 5′-terminal region of p53 mRNA provides an exceptional example in which synonymous (silent) mutations can affect functions of the encoded p53 protein (Table 2). Silent mutation at nucleotide position 201 (A>G, Leu 22), associated with chronic lymphocytic leukemia, impairs the p53 activity and regulation of the p53 translation mediated by HDM2 [39,79]. When treated with doxorubicin, H1299 cells expressing the mutated p53 fail to induce p53-dependent apoptosis in the presence of HDM2. The mutation at position 201 does not seem to change the secondary structure of the U180-A218 hairpin. However, it weakens the interaction of p53 mRNA with HDM2 by about 50% [61]. It has been suggested that this single silent mutation of p53 mRNA can affect the interaction with an oligonucleotide binding pocket in HDM2 by causing differences in the tertiary structure of the p53 mRNA [39]. Moreover, the mutation impairs localization of HDM2 to the nucleoli after genotoxic stress [61]. 

Two other silent mutations, one at nucleotide position 165 (C>T, Val 10) associated with colorectal carcinomas [80], and the other at position 243 (G>T, Pro 36) associated with non-melanoma skin cancer [81], weaken induction of the p53-dependent apoptosis. This effect could be caused by changes in the structure of the 5′-terminal region of p53 mRNA, and therefore, the mutations reduce its affinity for HDM2 [39]. Moreover, it has been observed that these mutations hamper the p53 expression and impair the accumulation of the p53 protein induced by DNA damage [39,61,79,80,81]. This suggests that these three mutations can affect the stability of the encoded p53 protein. 

Interestingly, artificial triple synonymous mutation introduced at nucleotide positions, 186 (A>T, Glu 17), 189 (A>C, Thr 18), and 192 (T>C, Phe 19), changes the structure of the U180-A218 hairpin in such a way that it causes an increase of p53 mRNA affinity to HDM2 [39]. Thus, this mutation does not change the level of apoptosis induced by p53, either in the presence or absence of HDM2. Moreover, the triple mutation stimulates synthesis and degradation of p53 in an HDM2-dependent manner under normal conditions. However, DNA-damaging stress, inducing the p53 mRNA-HDM2 interaction, does not result in an enhancement of the p53 degradation. This suggests that HDM2 may have access to the nascent p53 protein during HDM2-dependent translation, as shown by polysome pull-down assays of p53 [39]. 

Two artificial silent mutations have been introduced in p53 mRNA at nucleotide positions 180 (T>C, Ser 15) and 195 (A>G Ser 20). Both mutations correspond to the phosphorylation sites of p53 and have been proposed to be important elements in controlling the p53 activity [82,83,84]. In the U180-A218 hairpin, both mutations mimic the structural changes, similar to those caused by the alanine substitutions (Ser15Ala and Ser20Ala, respectively). Furthermore, both types of mutations (the silent mutations, as well as the alanine substitutions) have the same influence on the p53 function and both weaken the p53-induced apoptosis. This suggests that the nucleotide substitutions—rather than the changes in the amino acid sequence—are responsible for the observed effect on the p53 activity.

The single nucleotide polymorphism (SNP) at nucleotide position 120 (C>T) in the large hairpin domain in which the AUG1 codon is embedded is associated with human melanoma tumors [85]. The SNP has an important consequence on the translational control of p53, decreasing the cap-independent translation from 5′UTR of p53 mRNA (shown in bicistronic reporter assay) [40]. Moreover, the 5′UTR carrying this SNP shows a reduced affinity to PTB and impairs the enhancement of the IRES-mediated translation promoted by G2-M checkpoint, DNA-damaging stress, and oncogenic insult [40]. Interestingly, the C nucleotide at the 120 position in the 5′UTR of p53 mRNA is evolutionarily conserved. 

## 5. Conclusions and Perspectives

It has been shown that the 5′-terminal region of the p53 mRNA is highly involved in the translational regulation of the *p53* gene expression. The 5′-terminal region is the place of interactions with ribosomes and translational machinery canonical factors. The region’s structural features greatly modulate mRNA scanning. Additionally, it comprises the platform for binding non-canonical factors which specifically recognize unique RNA structural motifs. Moreover, the IRES elements present in the 5′-terminal region of p53 mRNA, together with ITAFs, promote cap-independent translation initiation, particularly when the canonical translation process is impaired. 

In all the above-mentioned functions of the 5′-terminal region of p53 mRNA, spatial folding plays an important, and even decisive, role. As presented in this review, the length of the 5′-terminal region variants, secondary structure elements which are formed within this mRNA region, and the plausibility of their unfolding, as well as structural environment of the initiation codons, can strongly modulate translation initiation. The ability of the 5′-terminal region of p53 mRNA to bind protein factors was also described, with special emphasis on the general principles that govern such RNA-protein interactions. Several of these proteins have a strong impact on the rate of scanning and on translation efficiency in in vitro assays and in selected cell lines, as well as under stress conditions. Thus, the structural features of the 5′-terminal region of p53 mRNA are very important for translation and for translation regulation mechanisms. 

Recent progress in RNA research has shown that further work is needed to fully explore the regulation mechanisms of the *p53* gene expression at the translational level. In particular, it is believed that mRNAs are refolded as they progress throughout their life-cycle, entering distinct subcellular compartments and associating with different proteins [86]. Thus, the determination of the structure of native p53 mRNA when it binds protein factors still remains a challenge. It might contribute to revealing the possible structural rearrangements of the mRNA in its cellular environment or may be induced by the binding of proteins that may be important for translation regulation. 

It has been estimated that as many as 10% of cellular mRNAs use IRESes for translation initiation [24,87,88,89]. However, better assays are still needed to identify and validate IRES elements [90]. Although p53 IRESes have already been well-characterized [91], they still need to be defined more precisely. It has to be taken into account that cellular IRESes are generally less structured than viral IRESes and can even be modular, meaning that the IRES activity is distributed throughout the 5′UTR [87]. The determination of the secondary structure of the 5′-terminal region of p53 mRNA [25,26] should help in further characterization of p53 IRESes. Very recently, it was reported that the expression of all known N-truncated p53 isoforms by re-initiation is mechanically feasible [92]. This possibility is important in light of p53 translational control and needs to be verified. 

Interestingly, it was also demonstrated that some proteins, which were thought to only interact with DNA, may interact with RNA to govern gene expression [93]. These observations broaden the spectrum of proteins which are potentially able to bind to RNA and influence gene expression. In the near future, it is likely that other proteins will be discovered that may interact with the 5′-terminal region of p53 mRNA and therefore affect the p53 translation.

Finally, it was shown that the most common post-transcriptional RNA modification, i.e., the generation of N6-methyladenosine (m6A), affects mRNA turnover and translation efficiency [94]. The m6A promotes the selective cap-independent initiation on certain mRNAs [95,96]. It is intriguing whether the 5′-terminal region of the p53 mRNA contains modified nucleotides, particularly m6A, and what function they play in translation, as well as in stress-induced conditions. 

## Figures and Tables

**Table 1:** The influence of cellular stress on p53 and Δ40p53 proteins synthesis.

Stress/Stress Factor	Site of Action	Response/Outcome	Reference
Ionizing radiation (IR)	5′-terminal region	Proteins that bind to the 5′UTR of p53 mRNA: RPL26 and nucleolin modulate p53 level and affect p53 induction after DNA damage	[55]
sodium nitroprusside or etoposide	5′-terminal region	Stress activates the binding of hnRNP Q to the 5′UTR of mouse p53 mRNA and regulates translation efficiency of p53	[46]
cisplatin and actinomycin D	HDM2 hairpin, within the first 101 nucleotides downstream of AUG1	Stress strongly enhances the binding of hnRNPC1/C2 to p53 mRNA	[58]
Doxorubicin	5′-terminal region	ATM-dependent phosphorylation of MDM2 leads to enhancement of p53 mRNA–MDM2 interaction and positively regulates p53 translation following DNA damage	[61]
Endoplasmic reticulum stress (ER)	HDM2 hairpin, within the first 120 nucleotides downstream of AUG1	Stress activates PERK kinase, which promotes mRNA translation and Δ40p53 synthesis, ER stress promotes selective oligomerization of Δ40p53	[76]
Glucose deprivation	IRES structures in 5′-terminal region	Stress activates SMAR1 protein, which binds to IRES element and results in elevation of p53 and Δ40p53 synthesis	[77]

**Table 2:** The influence of a single nucleotide polymorphism (SNP) and synonymous mutations within the 5′-terminal region of p53 mRNA on p53 functions.

Nucleotide Position/Mutation	Amino Acid Coded	RNA Structure	p53 mRNA-Protein Affinity	Translation and Degradation of p53	Effect on Apoptosis Induced by p53	References
201/A>G	Leu 22	Not changed	Reduction of HDM2 binding after genotoxic stress	Lower HDM2-mediated enhancement of p53 translation. Constant rate of p53 translation independently of HDM2 presence. p53 degradation inhibited during DNA damage	Inhibition of p53-dependent apoptosis upon doxorubicin treatment. Reduced in HDM2-dependent manner	[39,61,79]
165/C>T	Val 10	Changed	Reduction of HDM2 binding	Lower p53 expression level. Degradation inhibited during DNA damage	Reduced	[39,80]
243/G>T	Pro 36	Changed	Reduction of HDM2 binding	Degradation inhibited during DNA damage	Reduced	[39,81]
186/A>G	Glu 17	Changed	Reduction of HDM2 binding	Lower p53 expression level. Synthesis of p53 increased in HDM2-dependent manner	Not changed, even in the presence or absence of HDM2	[39]
189/A>C	Thr 18					
192/T>C	Phe 19					
180/T>C	Ser 15	Not changed	N.D.	N.D.	Reduced	[39]
195/A>G	Ser 20	Not changed	N.D.	N.D.	Reduced	[39]
120/C>T	-	N.D.	Reduction of PTB binding	Altered IRES activity	N.D.	[40]
